# The super-cycle of the global land-monsoon system

**DOI:** 10.1093/nsr/nwae092

**Published:** 2024-03-09

**Authors:** Yongyun Hu

**Affiliations:** Laboratory for Climate and Ocean-Atmosphere Studies, Department of Atmospheric and Oceanic Sciences, School of Physics, Peking University, China

## Abstract

The global land-monsoon system demonstrates super-cycles in both area and precipitation intensity in the Phanerozoic. The cyclicity of the global land-monsoon closely follows the supercontinent cycle, indicating the governance of continental configurations on the global land-monsoon over tectonic timescales.

The modern monsoon system consists of six regional monsoons [1]. These regional monsoons demonstrate coherently annual variations of precipitation and atmospheric circulation patterns, although they have different characteristics. Therefore, these regional monsoons are unified in the context of the global monsoon [1,2], which reflects the dominant mode of coherent annual variations of precipitation and atmospheric circulations in the tropics and subtropics.

A recent paper showed that over tectonic timescales both global land-monsoon areas and precipitation intensity are controlled by continental configurations, i.e. tropical continental area, continental fragmentation and latitudinal location of continents [3]. Specifically, the supercontinent Pangea was associated with a broad land-monsoon and weak precipitation, fragmented continents in the Cretaceous led to a smaller land-monsoon area and heavier precipitation, and reassembled continents in the Cenozoic resulted in a broader land-monsoon and weaker precipitation. It also shows that global land-monsoon area and precipitation intensity are highly anti-correlated. The correlation coefficient is as high as −0.87.

Here we show that the relationships of the global land-monsoon area and precipitation intensity with continental configurations are still held during the Phanerozoic, and that the evolution of the global land monsoon demonstrated super-cycles that closely followed the supercontinent cycle.

Figure 1a shows time series of global land-monsoon areas and precipitation intensity in the Phanerozoic. Global land-monsoon areas (blue line) show two troughs and two peaks. The first trough is approximately between 540 Ma and 330 Ma, and the second trough is between 160 Ma and 80 Ma. The two peaks are over 300–170 Ma and 70 Ma to present, respectively. Similarly, land-monsoon precipitation intensity of summer months also shows two peaks and two troughs, but highly anti-correlated with that of the land-monsoon areas. The correlation coefficient is −0.74. The anti-correlation and the governance of continental configurations to global land-monsoon area and precipitation intensity from 250 Ma to the present have been well addressed in ref. [3]. In the following, we focus on the relationships of land-monsoon area and precipitation intensity with continental configurations over the period from 540 to 260 Ma.

**Figure 1.**
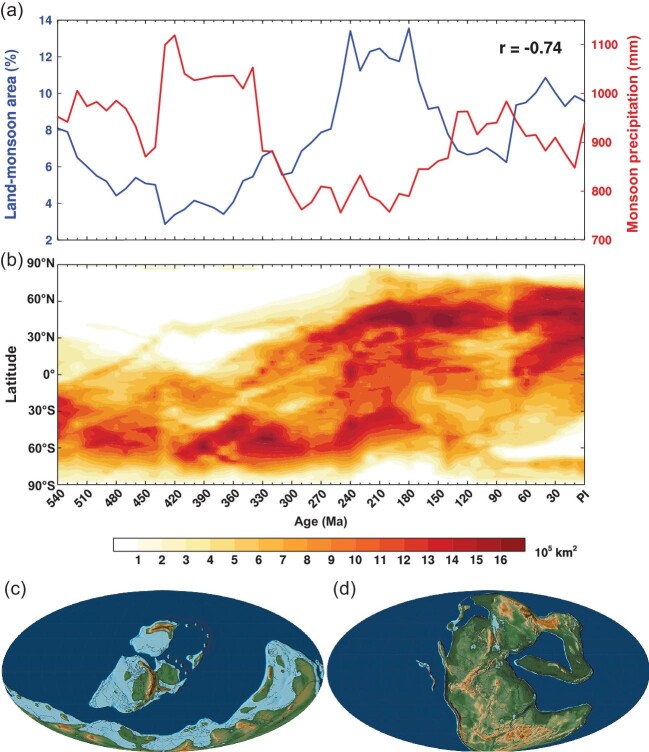
Super-cycles of the global land monsoon and continents in the Phanerozoic. (a) Time variations of global land-monsoon area (blue line) and precipitation intensity (red line). The left axis denotes the percentage of global land-monsoon area to Earth's surface area, and the right axis denotes precipitation of local summer months (May–June–July–August–September for the northern hemisphere, and November–December–January–February–March for the southern hemisphere), averaged over global land-monsoon domains. Unit: mm per 5-month. Here, the global land-monsoon domain is defined by the monsoon precipitation index proposed in ref. [1], i.e. monsoon domains are the regions where local summer-minus-winter precipitation exceeds 2 mm day^−1^, and local summer precipitation exceeds 55% of the annual total precipitation. Results are calculated from the simulation results in ref. [5]. (b) Latitudinal distribution of land areas and its evolution in the Phanerozoic, calculated from ref. [6]. Unit: 10^5^ km. Area weighting is considered for land area calculations at different latitudes. Paleogeography at (c) 420 Ma and (d) 240 Ma. The paleogeographic maps are from ref. [6].

In the Phanerozoic, continents have undergone the supercontinent cycle [4], assembling in the Paleozoic, breaking up in the Mesozoic and reassembling in the Cenozoic. Figure 1b shows the evolution of latitudinal distributions of land areas in the Phanerozoic. Over 540–360 Ma, continents were at the stage of assembling. Although Gondwana formed in this epoch, it was not fully assembled, and it was mainly located at the middle and high latitudes of the southern hemisphere. In the tropical region, there were only small pieces of continental plates, as shown by the paleogeographic maps of Fig. 1c. During this period, the tropical land area was small, and the associated land-monsoon area was small too (the first trough of the blue line in Fig. 1a). On the other hand, dispersive

tropical islands caused heavy monsoon precipitation (the first peak of the red line in Fig. 1a).

Over 350–270 Ma, continents gradually moved northward, assembled, and eventually formed the supercontinent Pangea (Fig. 1d). Associated with the continental evolution, the land-monsoon area became broader, and monsoon precipitation intensity became weaker, eventually forming the Megamonsoon in the Pangea era.

By extending analysis of our simulation results from 250 Ma back to 540 Ma, we show that the global land-monsoon system exhibits super-cycles in both area and precipitation intensity in the Phanerozoic, and that the cyclicity of the global land monsoon closely follows the supercontinent cycle. The results here further confirm that regional monsoons have coherent variations in both area and precipitation intensity over geological timescales, and that the variability of the global land-monsoon system is governed by continental configurations [3]. The governance of continental evolution is because of the fact that the global monsoon circulation is driven by land-sea thermal contrasts over tectonic timescales, rather than the mean climate state.

It has been shown that changes in carbon dioxide concentrations or surface temperatures have very minor effects on the super-cycle of the global land-monsoon system [3,7], although global mean surface temperatures fluctuated >10^º^C in the Phanerozoic. It is worth pointing out that topographic or orographic information was included in our simulations. However, topography has effects on regional monsoons, but is not a major factor in the global monsoon system. Finally, the simulation results need to be confirmed by geological proxy evidence in future studies.

## Data Availability

The data used in the present study are archived at https://doi.org/10.6084/m9.figshare.19920662.v1. Extended data are available at https://doi.org/10.1038/s41561-023-01288-y.
